# Diabetic Retinopathy: New Treatment Approaches Targeting Redox and Immune Mechanisms

**DOI:** 10.3390/antiox13050594

**Published:** 2024-05-12

**Authors:** Qi Tang, Francesco Buonfiglio, Elsa Wilma Böhm, Liyu Zhang, Norbert Pfeiffer, Christina A. Korb, Adrian Gericke

**Affiliations:** Department of Ophthalmology, University Medical Center, Johannes Gutenberg University Mainz, Langenbeckstrasse 1, 55131 Mainz, Germany; fbuonfig@uni-mainz.de (F.B.); elsawilma.boehm@unimedizin-mainz.de (E.W.B.); lzhang03@uni-mainz.de (L.Z.); norbert.pfeiffer@unimedizin-mainz.de (N.P.); christina.korb@unimedizin-mainz.de (C.A.K.)

**Keywords:** diabetic retinopathy, oxidative stress, reactive oxygen species, epigenetic changes, anti-VEGF drugs, corticosteroids

## Abstract

Diabetic retinopathy (DR) represents a severe complication of diabetes mellitus, characterized by irreversible visual impairment resulting from microvascular abnormalities. Since the global prevalence of diabetes continues to escalate, DR has emerged as a prominent area of research interest. The development and progression of DR encompass a complex interplay of pathological and physiological mechanisms, such as high glucose-induced oxidative stress, immune responses, vascular endothelial dysfunction, as well as damage to retinal neurons. Recent years have unveiled the involvement of genomic and epigenetic factors in the formation of DR mechanisms. At present, extensive research explores the potential of biomarkers such as cytokines, molecular and cell therapies, antioxidant interventions, and gene therapy for DR treatment. Notably, certain drugs, such as anti-VEGF agents, antioxidants, inhibitors of inflammatory responses, and protein kinase C (PKC)-β inhibitors, have demonstrated promising outcomes in clinical trials. Within this context, this review article aims to introduce the recent molecular research on DR and highlight the current progress in the field, with a particular focus on the emerging and experimental treatment strategies targeting the immune and redox signaling pathways.

## 1. Introduction

Diabetic retinopathy (DR) is a serious complication of diabetes mellitus and has become one of the leading causes of blindness in adults worldwide [1]. The current epidemiological studies have estimated that more than 500 million individuals worldwide grappled with diabetes in 2021 [2]. Remarkably, approximately one-third of the diabetic population potentially faces the threat of DR [3]. The projections suggest that, by 2045, the global diabetic population may surge to a staggering 700 million [4,5]. The hallmarks of DR encompass pathological transformations in retinal microvessels, including microaneurysms, intraretinal microvascular abnormalities (IRMAs) and neovascularization resulting in hemorrhages, hard exudates, and cotton-wool spots [6]. As the disease advances, it may give rise to severe complications such as macular edema, retinal detachment, vitreous hemorrhage, neovascular glaucoma, and, ultimately, irreversible blindness [7].

The pathophysiological mechanisms of DR are extraordinarily intricate, intimately intertwined with a myriad of microenvironmental shifts within the body. Initially, the heightened oxidative stress response to hyperglycemia triggers a cascade of effects, culminating in alterations to the signaling pathways, such as the protein kinase C (PKC) pathway, advanced glycation end products (AGEs) pathway, activation of glycolytic polyol pathways, and the overproduction of reactive oxygen species (ROS) [8,9]. These changes amplify the signaling from proliferative growth factors, such as the vascular endothelial growth factor (VEGF), resulting in the increased production and release of highly oxidatively reactive species, all contributing to the DR progression [10,11]. Second, the hyperglycemic environment in diabetes detrimentally affects the vascular endothelial cell function [12]. At the same time, changes in the retinal matrix and neural properties constitute pivotal components of the DR progression [13]. Additionally, inflammation and immune responses, including leukocyte adhesion, upregulation of chemokine expression, and heightened inflammatory factor production, play a pivotal role in DR development [14]. Furthermore, as molecular research on DR intensifies, potential targets and biomarkers, such as cytokines, chemokines, and growth factors, have come to the forefront. The influence of genetic and epigenetic variations on the onset and progression of DR has garnered substantial attention within the medical community [15].

Next to stringent blood sugar control and the optimization of the cardiovascular risk factors, the current treatment strategies for DR, such as stringent blood sugar and blood pressure control, laser photocoagulation, vitrectomy, intravitreal injections of anti-VEGF epidermal growth factor drugs, and intravitreal glucocorticoid therapy, all possess specific limitations and are unable to achieve the complete reversal of the disease [16]. In pursuit of overcoming these limitations, novel treatment avenues are explored. The purpose of the present article is to present the recent treatment approaches that target the redox and immune signaling pathways.

## 2. Molecular Mechanisms of Diabetic Retinopathy

Sustained hyperglycemia, oxidative stress, and other pathological factors trigger inflammatory responses within the retinal environment. This cascade of events initiates the activation, adherence, and infiltration of leukocytes, accompanied by the overexpression of inflammatory cytokines. Within this context, two significant pathophysiological phases of DR emerge, an initial non-proliferative stage followed by a subsequent proliferative stage. The former denotes an early disease phase marked by the loss of pericytes from the retinal capillaries, leading to the formation of acellular capillaries, increased vascular permeability, and disruption of the inner endothelial BRB [12]. The latter stage represents an advanced phase where fragile and tortuous blood vessels develop, ultimately culminating in the formation of fibrovascular epiretinal membranes, vitreous hemorrhage, and retinal detachment, ultimately resulting in visual impairment [12].

### 2.1. Oxidative Stress Induced by Hyperglycemia

Over the years, researchers have consistently identified elevated blood glucose levels as a pivotal factor in the pathogenesis of DR, causing retinal damage through a variety of direct and indirect mechanisms. In the following paragraphs, we present the most relevant pathomechanisms related to the oxidative stress occurring during DR.

#### 2.1.1. Mitochondrial Dysfunction

Mitochondria are the main source of intracellular ROS in humans [17]. Hyperglycemia induces mitochondrial dysfunction, exacerbating the electron leakage within the mitochondrial electron transport chain (ETC), resulting in the overproduction of ROS [18]. Specifically, chronic hyperglycemia triggers an overload of the reduced form of nicotinamide adenine dinucleotide (NADH) and the flavin adenine dinucleotide (FADH_2_) produced by the tricarboxylic acid cycle, subsequently leading to a high mitochondrial membrane potential and causing a slowdown of the ETC in complex III due to the challenging transfer of the electrons and protons carried by coenzyme Q downstream of the respiratory chain [19]. Moreover, in animal models of DR, elevated levels of the superoxide anion (O_2_^•−^) have been detected, accompanied by decreased expression of manganese (Mn) superoxide dismutase (SOD), also known as SOD2, one of the most relevant antioxidant enzymes in the human body, which is associated with retinal neuron apoptosis in DR [8]. In this context, utilizing a rodent model of diabetes, Du and colleagues reported an increased production of retinal O_2_^•−^ primarily related to mitochondria, with a smaller portion produced by NOX [20]. This research group further demonstrated in the same animal model an enhanced production of O_2_^•−^ specifically in the photoreceptor layer of both light-adapted and dark-adapted diabetic eyes [21]. Additionally, mitochondria also exhibit, under conditions of high serum glucose, mtDNA damage and morphological alterations, such as swelling, along with altered permeability [18,22].

Collectively, mitochondrial dysfunction is recognized as a significant pathophysiological driver capable of increasing ROS formation and thus responsible for the damaging events leading to the development of DR.

#### 2.1.2. Activation of the Protein Kinase C Pathway and Stimulation of NOX

The activation of PKC is recognized as one of the primary pathophysiological mechanisms driving DR. Hyperglycemia triggers the activation of PKC, notably the PKC-β isoform, by elevating the intracellular diacylglycerol (DAG) levels. Once PKC is activated, it modulates blood vessel permeability by the upregulation of VEGF, ultimately leading to retinal edema [19,23,24]. Consistent with these findings, an investigation conducted by Aiello and colleagues in diabetic patients demonstrated that the pharmacological inhibition of PKC-β via the oral administration of ruboxistaurin improved the diabetes-related retinal hemodynamic alterations [25]. PKC also influences the adhesion proteins, thereby affecting cell migration [8,24]. These changes may predispose the retina to neovascularization, a defining characteristic of advanced-stage DR. Importantly, several experimental studies have demonstrated that PKC can induce an increase in the NADPH oxidase (NOX) activity, favoring ROS generation in various vascular cells, including endothelial cells, dendritic cells, erythrocytes, smooth muscle cells, and pericytes [26,27,28,29]. Mechanistically, the production of O_2_^•−^ occurs through the addition of a single electron to molecular oxygen via the adenosine triphosphate (ATP) redox chain in mitochondria, catalyzed by the NOX in macrophages [30,31]. All NOX isoforms share a common domain comprising six transmembrane α helices chelating two iron-containing hemes, the flavin adenine dinucleotide (FAD), and the dehydrogenase domain (DH) of the NADPH substrate. Two electrons from the NADPH substrate are conveyed to FAD to form FADH_2_ [30]. FADH_2_ then transfers an electron to heme, subsequently transmitted to molecular oxygen to generate superoxide [30,32].

**Figure 1:**
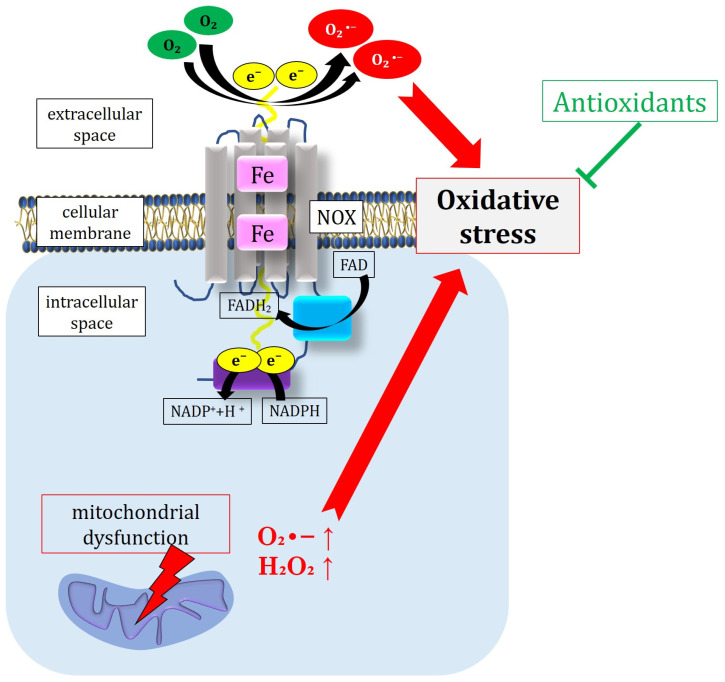
Overview of the molecular structure and mechanism of action of NOX, as well as regarding mitochondrial dysfunction and ROS generation. FAD: flavin adenine dinucleotide; Fe: heme group containing Fe atom; NADPH: nicotinamide adenine dinucleotide phosphate; NOX: NADPH oxidase; O_2_: oxygen; O_2_^•−^: superoxide anion; ROS: reactive oxygen species.

NOX is widely distributed in immune cells, with prominent isoforms including NOX1, NOX2, NOX3, NOX4, and NOX5. During the phagocytosis-mediated destruction of bacteria, NOX activation yields copious superoxide, subsequently converted to peroxide by SOD [33]. The excessive production of ROS overwhelms the antioxidant defense mechanisms, culminating in oxidative stress [8]. Figure 1 provides a schematic overview of the molecular structure and mechanism of action of NOX, as well as mitochondrial dysfunction and ROS generation, while antioxidant molecules work to counteract these pathomechanisms.

Two important enzymes of the vascular endothelium regulating the endothelial function in the retina include nitric oxide synthase (NOS) and cyclooxygenase (COX) [34]. NOS comprises three isomers: neuronal NOS (nNOS), inducible NOS (iNOS), and endothelial NOS (eNOS) [35]. The overproduction of ROS also triggers the activation of the retinal redox-sensitive transcription factor NF-κB [18]. In response, NF-κB amplifies the release of pro-inflammatory cytokines and the potent oxidant nitric oxide (NO). Under the catalytic influence of NOS, NO gives rise to a range of formidable oxidants [36]. These powerful oxidants can inflict cellular damage through the nitration of the tyrosine and phenylalanine residues in proteins [37]. In essence, the escalation of free radicals and the disturbance in the antioxidant system result in retinal cell DNA impairment, oxidative protein and lipid damage, functional perturbations in cells, and may even lead to the demise of retinal nerve cells. In addition, hyperglycemia triggers vascular endothelial dysfunction leading to impaired vasodilation [38]. These processes culminate in the eventual onset of retinal ischemia and neovascularization [10,11,39].

#### 2.1.3. Activation of the Polyol Pathway

An excess intracellular concentration of O_2_^•−^ triggers the enzymatic action of PARP, leading to the subsequent consumption of nicotinamide adenine dinucleotide (NAD⁺) [40]. Consequently, the activity of the glycolytic enzyme glyceraldehyde-3-phosphate dehydrogenase (GAPDH) significantly decreases, suppressing the glycolysis process [41]. Under these conditions, the polyol pathway experiences heightened activity and involves two principal steps [8]. First, glucose is reduced to sorbitol through the action of aldose reductase, utilizing nicotinamide adenine dinucleotide phosphate (NADPH) as a cofactor [42]. The sorbitol is subsequently oxidized to fructose by sorbitol dehydrogenase (SDH), converting the cofactor NAD⁺ to NADH [43]. As sorbitol has limited membrane permeability, it accumulates intracellularly, resulting in increased osmotic pressure and cellular damage, while the depletion of NAD⁺ correlates with the increased generation of ROS, due to the excess of NADH used as a substrate for NOX, contributing to the generation of intracellular O_2_^•−^ in retinal cells [8,44].

A study by Dagher and colleagues determined that blocking aldose reductase, the rate-limiting enzyme in the polyol pathway, protected against the early activation of the complement in the wall of retinal blood vessels in diabetic rats, as well as the later apoptosis of vascular pericytes and endothelial cells and the development of acellular capillaries [45]. The same study showed that both rat and human retinal endothelial cells display aldose reductase immunoreactivity, suggesting that an excess of aldose reductase activity can be a mechanism for human diabetic retinopathy [45]. Interestingly, the C106T polymorphism of the aldose reductase gene is linked with the severity of retinopathy in type 2 diabetes mellitus [46]. Moreover, the data suggest that the suppression of aldose reductase activity diminishes the neuronal apoptosis, glial response, and retinal neoangiogenesis in DR [47].

Additionally, fructose can be phosphorylated to fructose-3-phosphate and subsequently decomposed to 3-deoxyglucosone, eventually used as a precursor involved in the generation of advanced glycation end products (AGEs) through glycosylation [8,43]. Moreover, the increased compensatory action of the glucose monophosphate shunt leads to the excessive consumption of NADPH, thereby inducing a loss of the cofactors available for the synthesis of glutathione (GSH), ultimately exacerbating the oxidative stress due to a lack of endogenous antioxidant activity [18].

Altogether, the hyperglycemia-related activation of the polyol pathway dramatically alters the intracellular tonicity of the retinal capillaries, along with generating precursors of AGEs, culminating in an overabundance of ROS and the establishment of oxidative stress.

#### 2.1.4. Activation of the Hexosamine Biosynthetic Pathway

The hexosamine levels are elevated in the retinal tissues of diabetic patients [48]. The hexosamine biosynthetic pathway becomes hyperactive when there is excessive glycolytic intermediate fructose-6-phosphate that cannot be metabolized in glycolysis [49]. Under these circumstances, fructose-6-phosphate is converted to N-acetylglucosamine 6-phosphate (GlcNAc-6-P) by the enzymatic action of fructose-6-phosphate aminotransferase (GFAT), the rate-limiting enzyme in the hexosamine biosynthetic pathway [48,49,50]. GlcNAc-6-P is then converted to N-acetylglucosamine-1, 6-phosphate, and uridine 5′-diphospho-N-acetyl-d-glucosamine (UDP-GlcNAc) [48]. Consequently, in chronic hyperglycemia, the glucose flux through the hexosamine biosynthetic pathway increases, leading to enhanced production of UDP-GlcNAc, a pivotal donor substrate for the enzyme O-GlcNAc transferase (OGT). O-GlcNAcylation, mediated by OGT, is found to be elevated in diabetic animals and humans, causing the dysregulation of the signaling cascades and transcription [48,51]. For example, O-GlcNAcylation leads to modifications in transcription factor Sp1, which induces the expression of glucose-responsive gene plasminogen activator inhibitor-1 in vascular smooth muscle cells, contributing to DR [52,53]. Additionally, GlcNAc-6-P induces retinal pericyte loss and the generation of acellular capillaries by suppressing VEGFR2 and Ang2 in the normal retina [54]. Furthermore, elevated O-GlcNAcylation induces retinal ganglion cell death in diabetic murine models by affecting the NF-κB p65 subunit, while the enhanced O-GlcNAcylation of p53 is associated with increased retinal pericyte apoptosis, resulting in early DR vascular dysfunction [55]. Importantly, Kim et al. determined that the hexosamine pathway tends to increase retinal neuronal cell death by suppressing the neuroprotective effect of the insulin/Akt signaling pathway [56].

Critically, high levels of GlcNAc-6-P are known to cause an excess of ROS by reducing NADPH-dependent GSH production, leading to increased H_2_O_2_ levels [57,58], thereby affecting the mitochondrial respiration, further exacerbating oxidative stress, and promoting increased vascular permeability as well as neoangiogenesis [8].

#### 2.1.5. Formation of Advanced Glycation End Products

Elevated glucose levels give rise to the increased formation of AGEs within the polyol pathway. AGEs represent stable compounds formed via non-enzymatic catalysis, resulting from the covalent bonding of sugar molecules to proteins, lipids, or nucleic acids [59]. Once accumulated, AGEs activate various cell signaling pathways by binding with their receptor for advanced glycation end products (RAGE). This activation leads to oxidative stress, intensified inflammatory responses, and even influences gene expression, contributing to the damage in retinal cells and tissues [8,18,50].

The AGEs/RAGE axis induces NF-κB activation, thereby causing the pericyte apoptosis in the retina and overexpression of VEGF, which increases the vascular endothelial permeability [35]. The current literature reports that administering AGEs to normal rats upregulates RAGE and ICAM-1, leading to retinal hyperpermeability and leukostasis [60,61,62]. Moreover, hyperglycemia-related AGE formation induces the hyperactivation of the unfolded protein response and autophagy via endoplasmic reticulum stress, which also promotes pericyte apoptosis [63].

**Figure 2:**
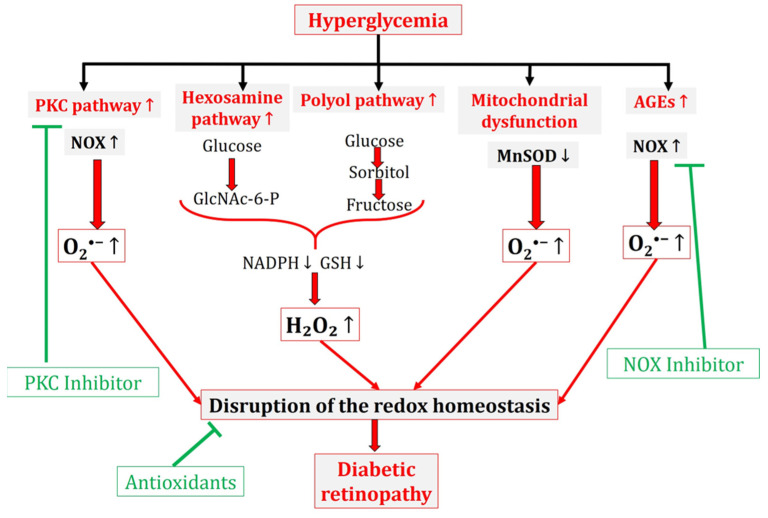
Schematic representation of the pathogenesis of DR leading to a disruption of the redox homeostasis in the eye. Hyperglycemia triggers oxidative stress responses through multiple pathways, ultimately leading to DR. In this context, PKC inhibitors, NOX inhibitors, and antioxidant agents antagonize the effect of oxidative stress. AGE: advanced glycation end products; GlcNAc-6-P: N-acetylglucosamine 6-phosphate; GSH: glutathione; H_2_O_2_: hydrogen peroxide; PKC: protein kinase C; NADPH: nicotinamide adenine dinucleotide phosphate; NOX: nicotinamide adenine dinucleotide phosphate oxidase; O_2_^•−^: superoxide anion; MnSOD: manganese superoxide dismutase.

Importantly, experimental studies have demonstrated that the binding of AGEs with RAGE triggers NOX, thereby increasing the intracellular ROS formation [64]. Furthermore, the existing literature highlights that AGEs can also induce ROS generation through the mitochondrial electron transport chain [65]. Conversely, ROS excess is involved in AGE formation and RAGE expression, establishing a positive feedback loop [8,66,67]. Figure 2 summarizes the main pathways leading to oxidative stress in DR.

### 2.2. Inflammatory Response

The pathogenesis of DR involves a multitude of factors, with the inflammatory response emerging as a crucial contributor to its progression [14]. In this context, it is noteworthy to describe two fundamental steps of the inflammatory events occurring in DR, the recruitment and activation of immune cells.

The capture and rolling of leukocytes are orchestrated by a family of cell adhesion proteins known as selectins. The three recognized selectin members (L-, E-, and P-selectin) exhibit similar structural topologies and bind to sialyl-Lewis^x^ carbohydrate ligands [68]. Under hyperglycemic conditions, vascular endothelial cells initiate the expression of a spectrum of adhesion molecules, and the selectin family of molecules is recognized to facilitate the initial rolling of leukocytes along the endothelium [69]. In particular, L- and E-selectin may play a role in the development of DR [69,70,71].

A distinct set of adhesion molecules intervenes to decelerate the leukocyte rolling and firmly anchor the leukocytes to the endothelial surface. The step of adhesion is mainly mediated by molecules of the immunoglobulin superfamily, such as the intercellular adhesion molecule ICAM-1 and the vascular cell adhesion molecule VCAM-1, expressed on endothelial cells. VCAM-1, in particular, has been found to be specifically associated with microvascular complications in DR [69]. The adhesion molecules expressed on the endothelial surface bind integrins, a class of transmembrane receptors expressed on leukocytes. The suppression of this interaction has been reported to prevent the formation of leakage in DR [72].

Once secure adhesion to the endothelial surface is achieved, adhesion proteins like vascular adhesion protein-1 (VAP-1), localized at the endothelial cell–cell junctions, facilitate the extravasation of the leukocytes between the endothelial cells along the intercellular junctions [73]. This process allows the leukocytes to traverse the vascular wall and infiltrate the retinal tissue, thereby inciting a localized inflammatory response.

Simultaneously, hyperglycemia can induce the activation of neutrophils, macrophages, and other immune cells while also triggering the release of the retinal redox-sensitive transcription factor NF-κB [14,74]. Notably, NF-κB has been demonstrated to instigate a pro-apoptotic program in response to the elevated glucose stress within retinal pericytes via the IL-1β pathway [75]. In this context, various pro-inflammatory cytokines are released, including tumor necrosis factor-α (TNF-α), along with chemokines such as monocyte chemoattractant protein-1 (MCP-1) [14,76]. These inflammatory factors can exacerbate the damage to vascular endothelial cells, disrupt the permeability of retinal vessels, and initiate retinal macular edema, which stands as a primary driver of the clinical manifestations of DR [14].

**Figure 3:**
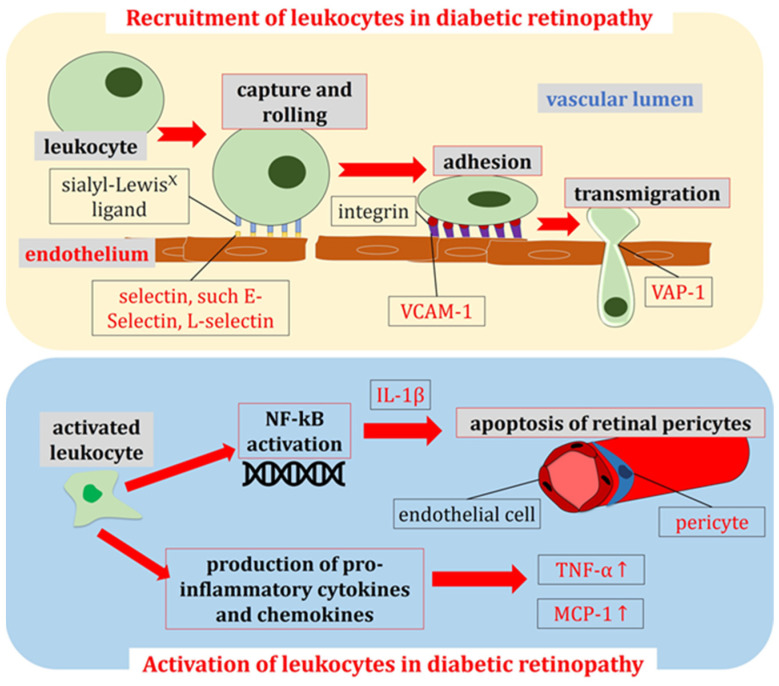
Illustration of the leukocyte recruitment along the endothelium and extravasation, as well as of the leukocyte activation in diabetic retinopathy. IL-1β: interleukin-1 beta; MCP-1: monocyte chemoattractant protein-1; NF-kB: nuclear factor kappa-light-chain-enhancer of activated B cells; TNF-α: tumor necrosis factor alpha; VAP-1: vascular adhesion protein-1; VCAM-1: vascular cell adhesion molecule-1.

Altogether, the inflammatory response of the retina is a key link in the pathogenesis of DR, and an in-depth understanding of it will help to find new therapeutic targets and optimize the treatment strategies. Figure 3 offers an overview on the processes of leukocyte recruitment and activation, leading to pericyte apoptosis and the production of pro-inflammatory cytokines during DR. Anti-inflammatory drugs act to antagonize these pathways, for example, by suppressing the action of specific cytokines.

### 2.3. Cellular and Molecular Changes

#### 2.3.1. Endothelial Cell Damage

DR is fundamentally a microvascular disease, and its primary pathophysiological mechanism revolves around the dysfunction of retinal vascular endothelial cells [77]. These crucial cells are responsible for maintaining vascular integrity and homeostasis, orchestrating the exchange of blood and nutrients, as well as the migration of the cells and molecules within the bloodstream [49]. In the diabetic state, persistent hyperglycemia, coupled with the systemic and local factors related to diabetes, such as arterial hypertension, dyslipidemia, elevated AGE levels, oxidative stress, and inflammatory agents, lead to impaired retinal vascular endothelial cell function, promoting the development of DR [39]. Specifically, dysfunctional vascular endothelial cells undergo various pathophysiological alterations, including increased permeability, decreased apoptosis and proliferation rates, as well as the increased expression of adhesion molecules and chemokines [14]. Consequently, the homeostasis of retinal microvessels is disrupted, resulting in vascular occlusion and increased permeability, the thickening of the capillary matrix, the development of macular edema, and the formation of new blood vessels, which are typical pathological features of DR [12].

The initial non-proliferative stage of DR is characterized by the loss of pericyte function, leading to microvascular abnormalities such as an increase in microvascular permeability and the apoptosis of endothelial cells. This contributes to the obstruction of retinal microvessels, exacerbates retinal edema, diminishes oxygenation capacity, and gives rise to non-perfused and hypoxic regions [78]. Hypoxia, in turn, stimulates the overexpression of growth factors such as VEGF, fostering the formation of new microvessels in the retina, which characterize the advanced proliferative phase [78]. However, owing to the damage inflicted by high blood sugar on endothelial cells, the structure of these newly formed microvessels often assumes an abnormal configuration, featuring thin walls, heightened permeability, and susceptibility to rupture and bleeding [79]. Hence, retinal edema and hemorrhage ensue, further exacerbating the visual impairment and serving as the primary pathological hallmark of advanced DR [80]. Hereby, strategies aimed at inhibiting VEGF, including the utilization of anti-VEGF antibodies and VEGF receptor antagonists, have become a cornerstone in the treatment of DR [81,82].

#### 2.3.2. Extracellular Matrix Remodeling

The retinal matrix and vascular basement membrane consist of a diverse array of cells and extracellular matrix (ECM) components, such as collagen IV, fibronectin, perlecan, and laminin, which can undergo structural and functional changes under persistent hyperglycemia [83]. The processes of glycosylation, oxidation, and the crosslinking of ECM proteins can occur during DR, leading to a thickening of the basement membrane [84]. These modifications not only impact the structural and functional aspects of the matrix but also influence the cellular behavior by activating a myriad of signaling pathways, including cell proliferation, migration, secretion, and apoptosis [85]. Hence, these pathogenetic events dramatically affect the vascular homeostasis and neural functionality of the retina. For example, collagen IV cross-linking can trigger the thickening of the retinal capillary walls, impeding blood permeability and fluidity, ultimately contributing to edema [83]. Furthermore, changes in the matrix proteins can influence cell adhesion and migration, thereby promoting neovascularization [84,86]. Additionally, high levels of matrix metalloproteinases (MMPs), which belong to the endopeptidase family responsible for ECM degradation or remodeling, have been found in DR, and appear to be implicated in the retinal revascularization during the late stages of DR [87,88,89].

### 2.4. Retinal Neurodegeneration

Recent studies have unveiled the role of retinal neurodegeneration in the pathogenesis of DR [90,91]. Several investigations have demonstrated progressive retinal thinning and visual dysfunction in individuals affected by diabetes, prior to the occurrence of DR or in the very early stages of DR [92], with retinal imaging revealing vascular remodeling and choroidal changes [93,94,95,96,97,98]. Additionally, studies on animal models of diabetes have shown that diabetes directly impacts the retinal neural and glial cells, supporting the notion that diabetic retinal neurodegeneration occurs early in the disease [99,100,101,102,103,104,105,106].

Pathogenetically, damage to retinal neurons manifests as the degeneration of cell bodies and synapses, resulting in the impaired transmission of visual signals. This disruption of the synaptic connections among the retinal nerve cells affects the retinal neurovascular coupling, contributing to the atrophy and occlusion of retinal blood vessels [90,107]. This process stands as a significant cause of the early visual impairment in DR. Subsequently, the survival and functionality of retinal neurons are contingent upon the blood and oxygen supply from retinal blood vessels [108]. In DR, the dysfunction of vascular endothelial cells and the increase in ROS levels result in retinal ischemia and hypoxia, leading to ischemic injury to neurons [90,91]. Neuronal damage, in turn, exacerbates the progression of DR by impairing the light sensing and signal conduction within the retina, thereby leading to diminished vision. Moreover, neuronal damage can precipitate structural alterations in the retina, including the detachment of the nerve fiber layer and thinning of the ganglion cell layer, further intensifying the course of the disease [13].

Retinal glial cell dysfunction may be intricately linked to the development and amplification of the retinal inflammation in DR [109]. The retina houses various types of glial cells, including astrocytes, Müller cells, and microglia. Astrocytes are the largest subtype of glial cells and are responsible for blood–brain barrier formation, the regulation of blood flow, and the modulation of synapse formation and transmission [110]. Müller cells are radial cells that extend through all the retinal layers, further accounting for approximately 90% of the retinal glia population, with fundamental trophic activity [111]. Microglia belong to the immune system and represent the resident tissue macrophages, crucial for retinal homeostasis and recovery from damage [112]. Injury to these cellular subpopulations compromises their roles in neuroprotection, blood–brain barrier maintenance, and antioxidant defense. Under the stress of hyperglycemia, microglia and Müller cells become activated, resulting in the heightened production and release of inflammatory molecules such as TNF-α, IL-6, MCP-1, and VEGF, accompanied by increased secretion of MMPs [113]. In a hyperglycemic environment, Müller cells generate greater quantities of inflammatory factors like NO and COX [91,114]. Moreover, this environment may induce Müller cells to augment intermediate filament synthesis, culminating in the formation of a dense fibrotic layer enveloping the retina and impeding its pathways [91]. Furthermore, the aforementioned neurodegenerative changes intersect with various responsive processes in the high-glucose environment, although the specific mechanisms warrant further investigation.

**Figure 4:**
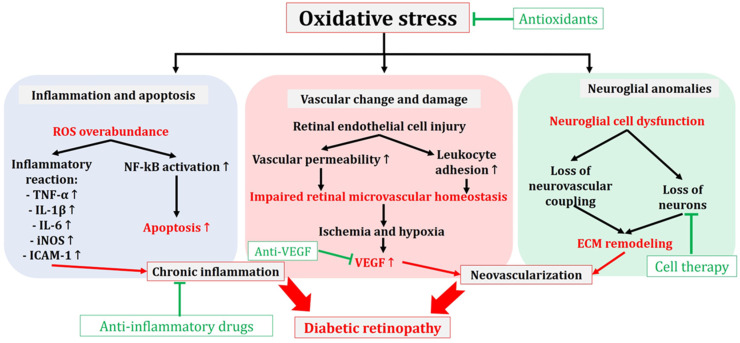
Oxidative stress activates various pathogenetic events. In terms of blood vessels, retinal endothelial cell damage, changes in retinal blood flow pattern, and glial dysfunction will all promote increases in vascular permeability, ischemia, and hypoxia and lead to insufficient perfusion, and eventually induce neoangiogenesis. Parallelly, processes of inflammation and apoptosis lead to chronic immune reactivity and to loss of neural cells. ECM: extracellular matrix; ICAM-1: intercellular adhesion molecule-1; IL-1β: interleukin-1 beta; IL-6: interleukin-6; iNOS: nitric oxide synthases; NF-κB: nuclear factor kappa-light-chain-enhancer of activated B cells; ROS: reactive oxygen species; TNF-α: tumor necrosis factor alpha; VEGF: vascular endothelial growth factor.

Figure 4 illustrates the pathogenetic events triggered by the oxidative stress during DR, comprising inflammation, apoptosis, neoangiogenesis, and neuroglial anomalies. These processes are mitigated by antioxidants, anti-inflammatory drugs, anti-VEGF agents, and the strategies of cell therapy.

### 2.5. Genetic and Epigenetic Alterations

While internal environmental factors such as glycemic control, blood pressure, and lipid levels have an important impact on the onset and progression of DR, the significant differences in the susceptibility and severity of DR among individuals suggest that genetic factors also play a role in this process [15].

For example, numerous studies have identified associations between gene polymorphisms and the occurrence and progression of DR. For instance, the polymorphisms in genes such as VEGF, involved in angiogenesis, TNF-α, related to inflammation, and eNOS, associated with endothelial cell function, have been closely linked to DR development [115]. In addition, the genes implicated in diabetic complications, such as AKR1B1, the receptor for AGE (RAGE), and eNOS genes, have shown associations with DR [116]. These genetic variations may impact the expression or activity of related proteins, subsequently influencing the DR pathogenesis [23].

In parallel, epigenetic modifications also play a role in the course of DR. The persistent adverse effects of hyperglycemia on the progression of diabetic complications, even after achieving glycemic control, demonstrate a phenomenon known as the “metabolic memory” of diabetes, potentially attributed to the epigenetic changes in specific cells under diabetic conditions [15]. Therefore, the significant occurrence of epigenetic modifications in the development and progression of metabolic memory during DR becomes a substantial factor in the pathophysiology of this disorder [117].

In fact, in the hyperglycemic environment, various mechanisms, including DNA methylation, histone modification, non-coding RNA regulation, and others, can modulate the gene expression without altering the DNA sequence [118]. For example, studies have revealed epigenetic modifications of *SOD2* during DR development. The methylation of lysine 20 of histone H4 (H4K20) at the promoter and enhancer regions of *SOD2* increases in hyperglycemic conditions, a finding supported by observations in the retinas of human donors with DR [119,120]. DNA methylation primarily occurs on cytosine–phosphate–guanine (CpG) islands, and elevated blood sugar levels activate DNA methyltransferases (DNMTs). S-adenosyl-l-methionine (SAM), a common methyl donor for DNMTs, plays a role in this process. Oxidatively damaged methyl-CpG sequences induce epigenetic changes in the chromatin organization by disrupting the interaction between methyl-CpG sites and the binding domain of methyl-CpG-binding protein 2 (MeCP2) [15]. Histone modifications, including methylation, acetylation, acylation, and ubiquitination, can either activate or repress genes depending on the number of methyl groups added, the specific histone residue modified, and its location in the N-terminal region of H3 or H4. Histone acetylation predominantly occurs on lysine residues in the amino-terminal tail and is regulated by histone acetyltransferases (HATs), whose activity is responsive to the intracellular high-glucose environment [121].

Furthermore, chromatin modifications are not solely governed by these epigenetic marks but also influenced by non-coding RNAs. Both small non-coding RNAs (microRNAs) and long non-coding RNAs (lncRNAs) exhibit altered expression in diabetic patients. MiRNAs, for example, can repress transcription or degrade target mRNAs by binding to them. The research indicates that the downregulation of miRNAs contributes to the elevation of VEGF and NF-κB in the DR progression [15]. Nonetheless, it is important to note that, while these molecular mechanisms shed light on the pathogenesis of DR, they do not provide a complete explanation of the entire pathological process. Therefore, further research is necessary to comprehensively understand the pathogenesis of DR and to identify more potential therapeutic targets.

## 3. Ongoing and Cutting-Edge Research in DR Care

Over recent decades, the advancements in research have substantially deepened our understanding of numerous eye diseases. Notably, significant progress has been achieved in elucidating the molecular pathomechanisms underlying debilitating ocular disorders [122,123,124,125]. Regarding DR, these breakthroughs transcend the mere comprehension of its molecular mechanisms, extending to the identification of novel therapeutic, diagnostic, and preventive strategies that have transitioned into clinical trials. These include promising modalities such as genetic therapy and stem cell therapy, offering new avenues for treatment and management.

### 3.1. Discovery of Biomarkers

While the diagnosis and clinical staging of DR primarily rely on fundus examination, this approach is constrained by equipment and technology limitations and is unable to achieve early detection and precise staging. Consequently, the identification of DR biomarkers carries significant clinical value. Diabetes triggers systemic metabolic disturbances, affecting protein, lipid, and nucleic acid metabolism, leading to changes in metabolites’ concentrations in body fluids. For instance, increased levels of glycosylated hemoglobin (HbA1c), an indicator of hyperglycemia, are directly associated with the risk of DR [126]. Additionally, the concentrations of VEGF and ICAM-1 in the serum and vitreous fluid of diabetic patients exhibit notable elevation [127]. TNF-α, a potent inflammatory cytokine, plays a significant role in the DR pathology, making it a proposed biomarker candidate [128,129]. Recently, novel biomarkers such as retinal nerve growth factor (NGF) and retinal neuron-specific neurofilament light chain (NFL) have been discovered, and changes in their concentrations can predict the status of retinal neuronal injury [77,130]. Monitoring the changes in these biomarkers offers the potential for early DR diagnosis and the monitoring of disease progression, representing a clinically valuable approach.

Presently, many researchers are considering biomarkers in the development of new drugs. For example, anti-VEGF drugs have been utilized to treat macular edema and neovascularization in DR [131].

### 3.2. Advances in Molecular Therapy

Molecular therapy predominantly targets the key signaling pathways implicated in DR, leading to the development of numerous innovative therapeutic strategies.

#### 3.2.1. Anti-VEGF Molecules

Anti-VEGF drugs are specifically formulated to impede the formation of new blood vessels by counteracting the effects of VEGF, with a primary focus on reducing the antipermeability to alleviate fluid accumulation and edema. Among these drugs, ranibizumab stands out as the sole approved one for proliferative diabetic retinopathy. This synthetic antibody binds to VEGF, disrupting its interaction with the receptors and thereby inhibiting angiogenesis [132]. Additional anti-VEGF drugs have been applied for diabetic retinopathy, including aflibercept, faricimab, conbercept, bevacizumab, and brolucizumab. Notably, conbercept and brolucizumab have gained approval for intravitreal treatment in diabetic macular edema [133]. Faricimab, in particular, emerges as a promising therapeutic agent for diabetic macular edema. This drug combines anti-VEGF therapy with the inhibition of angiopoietin-2, resulting in reduced inflammation and improved vascular permeability, as demonstrated in a murine model of retinal neovascularization and ischemia/reperfusion following dual inhibition [134,135]. The primary objective of anti-VEGF use in this context is to treat DR to prevent complications and disease progression [136]. However, despite the evident efficacy of anti-VEGF drugs for numerous DR patients, challenges persist. Some individuals exhibit inadequate responses to treatment, and the inconvenience of regular intraocular injections remains a notable concern.

Numerous clinical trials have been conducted in recent years on the use of anti-VEGFs to manage DR [137,138]. Specifically, recent investigations have evaluated the efficacy and safety of various anti-VEGFs, such as ranibizumab, aflibercept, and bevacizumab, in managing DR, reporting benefits in improving patient outcomes [131,139,140,141,142,143,144,145]. Interestingly, recent studies, including network meta-analyses, have shed light on the effectiveness of biologic drugs by comparing dedicated clinical trials for treating DR. Fallico and associates reported limited evidence of comparable efficacy in terms of the neovascularization regression between pan-retinal photocoagulation and anti-VEGF therapy alone or in combination with pan-retinal photocoagulation. However, better visual outcomes were associated with the use of anti-VEGFs [146]. Consistent with these findings, another network meta-analysis by Zhang and collaborators compared the efficacy of ranibizumab, ranibizumab + laser, aflibercept, laser alone, and pan-retinal photocoagulation, demonstrating the best visual score improvement with ranibizumab alone [147]. A recent study by Wang et al. focused on determining the optimal timing for administering anti-VEGF, specifically conbercept, as an adjuvant to pars plana vitrectomy for treating DR, revealing that preoperative intravitreal conbercept is an effective adjuvant [148].

#### 3.2.2. Anti-Inflammatory Drugs

Nonsteroidal anti-inflammatory drugs (NSAIDs) function by inhibiting COX enzymes, thereby reducing the synthesis of inflammatory mediators, including prostaglandins. Certain ophthalmic-specific NSAIDs have proven to be effective in mitigating macular edema and enhancing vision. Notable examples include ketorolac, a potent COX-1 inhibitor, and bromfenac, a potent COX-2 inhibitor [149,150,151,152].

The main pathways regulating the signal transmission for many cytokines are the Janus kinase (JAK) or signal transducer and activator of transcript (STAT) signaling pathways. The cytokines and growth factors involved in diabetic retinal complications, such as VEGF, IL-6, and IL-17a, are all controlled by these pathways [153,154]. The experiments involving JAK1 inhibitors, such as Tofacitinib, in mice with type II diabetes revealed increased expression of PJAK1 in the retina, suggesting that JAK inhibitors can enhance blood–retina barrier function, subsequently reducing the blood vessel penetration and inflammation. These findings offer new potential strategies for treating diabetic retinal complications [155].

The inflammatory cytokine IL-6 is widely recognized for promoting increased vascular endothelial monolayer permeability and disrupting the vascular barrier in inflammatory diseases [156]. Studies indicate that the channels related to DR will amplify and prolong the effects of the inflammatory cytokine IL-6 under high-glucose conditions [157]. A meta-analysis conducted by Yao and associates determined that IL-6 is generally elevated in patients with DR, probably being further linked with the severity of the disease [158].

Mechanistically, the IL-6 signaling pathway is classically activated through the binding of IL-6 with its membrane-bound IL-6 receptor, known as “classical signaling” [159]. However, IL-6 signaling is also observed in cells lacking the membrane-bound IL-6 receptor due to the presence of a soluble IL-6 receptor (sIL-6R), termed “trans-signaling” [159]. The current literature suggests that IL-6 classical signaling is predominantly anti-inflammatory, whereas IL-6 trans-signaling elicits the pro-inflammatory effects of IL-6 [160,161,162]. Interestingly, Robinson and colleagues demonstrated in a murine model of early DR that the inhibition of Interleukin-6 trans-signaling prevents oxidative stress [163].

The pleiotropic effects of IL-6 are mainly observed in

(1). The activation of vascular endothelial cells leading to the production of IL-6, IL-8, monocyte chemoattractant protein-1 (MCP-1), intercellular adhesion molecule-1 (ICAM-1), and C5a receptor, as well as the induction of vascular endothelial cadherin disassembly [164].
(2). Binding to the receptor complex, allowing gp130 to activate the tyrosine kinases of the JAK kinase family, including Jaks, Jak1, Jak2, and Tyk2 [159]. The phosphorylation of STAT3 by JAK promotes the growth arrest and differentiation of macrophages, resulting in the persistent loss of endothelial barrier function [165,166]. In this context, an in vitro study by Valle and colleagues in human retinal endothelial cells demonstrated that a blockade of the IL-6 trans-signaling prevents inflammation and endothelial barrier disruption, decreasing the phosphorylation of STAT3 [167].
(3). IL-6 stimulation also activates the mitogen-activated protein kinase (MAPK) cascade and the phosphatidylinositol-3-hydroxykinase (PI3K) cascade by binding to the signaling molecule protein, tyrosine phosphatase 2 (SHP2) [168].

**Figure 5:**
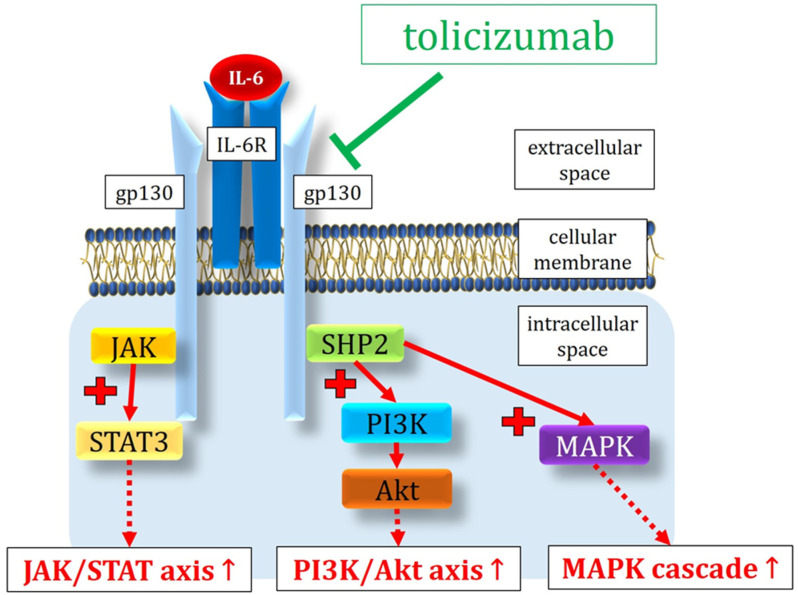
The IL-6 signaling pathway and the mechanism of action of tolicizumab. Through a receptor composed of signal transduction subunit glycoprotein 130 (gp130) and the auxiliary transmembrane protein IL-6 receptor (IL-6R), IL-6 activates the JAK/STAT axis, while the MAPK cascade and the PI3K/Akt axis are activated by the domain SHP2. Tolicizumab antagonizes the subunit gp130. Akt: Ak strain transforming (also known as protein kinase B, PKB); IL-6: interleukin-6; IL-6R: IL-6 receptor; gp130: glycoprotein 130; JAK: Janus kinase; MAPK: mitogen-activated protein kinase; PI3K: phosphoinositide 3-kinases; SHP2: src homology phosphotvrosvlphosphatase 2; STAT: signal transducer and activator of transcription.

The humanized IL-6 receptor antibody, tocilizumab, exhibits the capability to block the IL-6-mediated signaling pathways by inhibiting the binding of IL-6 to soluble and membrane-bound forms of the IL-6 receptor. This provides new insights for the study and treatment of the complications associated with DR [168]. Figure 5 provides a schematic illustration of the main molecular pathways activated by the binding between IL-6 and its receptor.

#### 3.2.3. Antioxidant Therapy

As previously discussed, oxidative stress and endothelial dysfunction play pivotal roles in promoting the complications related to DR [169]. ROS, particularly O_2_^•−^, adversely affect the vasorelaxation and endothelial protective factor NO by uncoupling eNOS. In a hyperglycemic environment, the increased electron leakage in the mitochondrial electron transport chain leads to ROS overproduction, triggering an oxidative stress response involving various biomarkers. The NOX family serves as the enzymatic source of oxidative stress, intensifying the endothelial inflammation and cellular damage. Consequently, investigating various antioxidant drugs becomes a promising therapeutic avenue. Antioxidant enzymes, including catalase, superoxide dismutase, and glutathione peroxidase, along with their cofactors, form the primary cellular antioxidant defense system. Effective antioxidants, such as coenzyme Q10, have demonstrated potential in ameliorating visual impairment in DR [18]. Many plants are rich in antioxidants, such as flavonoids, polyphenols, and carotenoids, with compounds like lutein showing promise in mitigating the neurodegenerative effects caused by the local oxidative stress in the diabetic retina [18].

Sulodexide, a glycosaminoglycan mixture composed of low-molecular-weight heparin and dermatan sulfate, exhibits a diverse range of biological effects on the vasculature. These include antithrombotic, profibrinolytic, anti-inflammatory, and endothelial protection [170,171,172]. In an oxygen-induced retinopathy (OIR) mouse model, the antiangiogenic effect of sulodexide was evident, enhancing the glycocalyx of diabetic retinal arterioles, reducing the vascular permeability, and inhibiting the retinal neovascularization in vivo [173]. The glycocalyx is a layer of adhesive material that covers the lumen of vascular endothelial cells. It plays a crucial role in maintaining the dynamic balance of the vasculature, controlling vascular permeability, microvascular tone, preventing microvascular thrombosis, and regulating leukocyte adhesion. The hyaluronate glycosaminoglycan, a main component of the glycocalyx, is essential for maintaining the endothelial barrier properties of plasma macromolecules [174]. Experiments involving isolated pig retinal arterioles exposed to sulodexide and hyperglycemia demonstrated that sulodexide protects retinal arterioles from the oxidative stress and endothelial dysfunction induced by hyperglycemia [38]. Remarkably, sulodexide prevented the overexpression of the oxidative stress-related proteins NOX 4 and NOX 5 in retinal arterioles exposed to a high glucose concentration. These findings suggest that sulodexide may prevent the excessive ROS production and endothelial dysfunction in the retinal arterioles exposed to hyperglycemia [38]. In aged human retinal endothelial cells under hyperglycemic conditions, sulodexide reduced the IL-6 and VEGF-A secretion, decreased the expression of the senescent protein p53 gene, and increased the transendothelial resistance. The reduced transendothelial resistance values during hyperglycemia reflect glycocalyx damage and increased vessel wall permeability. This evidence indicates that sulodexide delays the impact of a high glucose concentration on human retinal endothelial cell damage and aging [171]. Furthermore, sulodexide, with its high oral bioavailability and demonstrated tolerability and safety in clinical studies [170], represents a promising therapeutic option in the treatment of diabetic retinopathy.

Resveratrol is a non-flavonoid polyphenolic compound rich in red wine, which exerts antioxidant, anti-inflammatory, and protective cardiovascular effects [175,176]. This molecule was shown to inhibit the (MAPK)/ERK1/2 cascade to protect retinal ganglion cells and blood vessels from hydrogen peroxide-induced apoptosis [176,177,178]. In the context of a high glucose concentration, resveratrol also mitigates the oxidative stress damage in retinal capillary endothelial cells by activating the AMPK/Sirt1/PGC-1α pathway [35,179].

The recent research has focused extensively on resveratrol in DR. Notably, resveratrol treatment in diabetic rats reduces retinal cell apoptosis by regulating the expression of RNA-dependent-protein-kinase associated protein X (RAX)/phosphorylated RNA-dependent-protein-kinase (RAX/P-PKR) [180]. Moreover, resveratrol inhibits the expression of VEGF, ACE, and matrix metalloproteinase (MMP)-9 mRNA, protecting the retina [10,181,182]. Some cytological experiments have also demonstrated the protective effect of resveratrol on the retina. Resveratrol treatment can significantly reduce the expression levels of acetylated NF-κB p65 and p53 proteins, thereby inhibiting oxidative damage, inflammation, and apoptosis [183]. In addition, resveratrol intervention in high-glucose environments prevents oxidative stress and phenotypic modifications of the retinal endothelial cells by inhibiting the PKC pathway, thus attenuating endothelial-to-mesenchymal cell transformation (EndMT) and reducing the occurrence of DR-related fibrosis [184].

Diabetes can lead to the transcriptional upregulation of many genes related to the intrinsic apoptosis pathway. As an antioxidant, trans-resveratrol can upregulate intrinsic apoptosis pathway-related proteins, such as caspase-9, caspase-3, and ERK1, and normalize the MAPK signal transduction in diabetic rats [185]. Furthermore, resveratrol demonstrates the capacity to diminish vascular lesions and curb the activity of NF-kB and TNF-α [186]. Intriguingly, the oxidative stress induced in the RPE by a high-glucose environment can hinder the retinoic acid metabolic pathway, yet the short-term intervention with trans-resveratrol demonstrates an ameliorative effect. However, prolonged exposure yields contrasting results [187]. Additionally, under resveratrol intervention, the retinal cells in a high-glucose environment exhibit the inhibition of VEGF, TGF-β1, COX-2, IL-6, and IL-8 accumulation, as well as the suppression of PKC activation and the degradation of connexin 43, along with gap junction intercellular communication, in a dose-dependent manner. Resveratrol also significantly mitigates the retinal vascular permeability and VEGF levels in diabetic rats while improving the diabetes-related indicators. This effect is speculated to occur through AMPK activation, leading to reduced VEGF accumulation [188], and by inhibiting transforming growth factor-β2 (TGF-β2)-induced endothelial-to-mesenchymal transition, thereby suppressing the VEGF expression in adult RPE cells [189].

Additionally, resveratrol activates the sirtuin (SIRT) pathways in DR treatment, modulating the inflammatory responses, insulin sensitivity, and sugar metabolism [190]. It effectively prevents high glucose-induced senescence in endothelial cells by upregulating SIRT3, SIRT4, and SIRT5 expression [179].

Paraoxonase (PON), an enzyme associated with the high-density lipoprotein (HDL) and low-density lipoprotein (LDL), possesses antioxidant properties and has been implicated as a potential risk gene for DR lesions. Resveratrol restores the PON1 expression and activity, indicating its potential through PON1 to mitigate visual DR lesions [191].

Despite its hydrophilic nature, resveratrol can enter the retina. Efforts to improve its oral administration include microencapsulation in polymers, enabling efficient delivery near the nucleus of human retinal pigment epithelial cells [192]. Moreover, gold nanoparticles synthesized from plant extracts have shown promise in reducing the inflammatory markers in diabetic rats, offering new avenues for the effective intraocular delivery of resveratrol [193].

Betulinic acid emerges as a bioactive compound wielding potent anti-lymphocytic leukemia properties alongside anti-inflammatory, antioxidant, and anti-reperfusion injury capabilities [194]. Our experimental investigations have substantiated that betulinic acid serves as a protective agent, averting the retinal ganglion cell loss and axonal damage in the optic nerve while concurrently curbing the generation of ROS in retinal vessels following I/R. This dual action not only safeguards against the retinal damage resulting from the I/R events but also underscores the potential of betulinic acid as a therapeutic intervention for preserving retinal health [195].

The pentacyclic triterpenoids represented by betulinic acid have significant regulatory effects on glucose absorption and uptake, insulin secretion, diabetic vascular dysfunction, and retinopathy [196]. Furthermore, they prevent apoptosis and attenuate the ROS production in human Müller cells treated with glutamate, potentially through the modulation of the Akt/MAPK signaling cascade [197]. The interaction of betulinic acid with α-glucosidase suggests a potential therapeutic avenue for managing postprandial hyperglycemia, a significant risk factor for cardiovascular disease in patients with diabetes mellitus and DR [198]. Thus, the study of betulinic acid presents a promising direction for the therapeutic research in DR.

High blood glucose levels promote the production of AGEs with following activation of the RAGE in retinal cells to induce ROS production. The RAGE has been reported to accelerate the cell decay in bovine retinal pericytes by the induction of oxidative stress [35]. Furthermore, a close association between a high-fat diet, obesity, insulin resistance, and diabetes, where a high-fat diet induces an oxidative stress response in the ocular arteries, has been found. This is achieved by RAGE-induced ROS production, suggesting that a high-fat diet triggers hypercholesterolemia and hyperglycemia, culminating in ROS generation and endothelial dysfunction in ocular arteries in mice [199]. The activation of the RAGE initiates a cascade of events, including the interaction between its ligand and the extracellular domain of the RAGE. This leads to the production of ROS, ERK, JAK/STAT, and MAPK cascade reactions, as well as the activation of transcription factors (AP-1 and NF-κB) and the Rho family [200]. Consequently, drug studies targeting the RAGE, such as RAGE antagonists and blockers binding to RAGE ligands, show promise. The emerging research directions, such as the small molecule RAGE inhibitor TTP488 (azeliragon), are considered promising therapeutic approaches to inhibit RAGE-mediated signaling [30,201,202].

The inhibition of certain NOX isoforms associated with DR has also demonstrated efficacy in preventing DR progression [203]. For example, lovastatin or carotenoids inhibiting NOX-4 have been shown to reduce ROS generation and VEGF overexpression [204].

Nuclear factor erythroid 2-related factor 2 (Nrf2) plays a pivotal role in regulating the antioxidative defense system. Oxidative stress arises from an imbalance between ROS generation and the antioxidant defense system, and the dysregulation of Nrf2 is linked to oxidative stress-related diseases, including diabetic retinopathy [18]. The modulation of Nrf2, as exemplified by fenofibrate, has proven effective in ameliorating diabetic retinopathy by reducing the ROS formation, retinal leukostasis, and vascular leakage [205]. In addition, maslinic acid has demonstrated protective effects against streptozotocin-induced diabetic retinopathy through the upregulation of Nrf2 and downregulation of NF-κB [206]. Other agents, such as tricin or urolithin A, have also shown positive impacts on diabetic retinopathy [207,208], highlighting Nrf2 as a promising therapeutic target in the management of diabetic retinopathy.

Sulforaphane (1-isothiocyanate-4-methylsulfinyl butane) serves as an activator of Nrf 2 and is prominently present in cruciferous plants such as broccoli, Brussels sprouts, and cabbage [209].Recognized for its epigenetic, antioxidant, and anti-inflammatory effects, sulforaphane holds promise for neuroprotection in various retinal disease models [210]. Notably, the lipophilic nature of sulforaphane enables it to traverse cell membranes, cross the blood–brain barrier through transmembrane diffusion, and activate Nrf2 signaling in vivo. This attribute, coupled with a safer route of administration, suggests greater clinical potential [211].In a recent experiment involving diabetic rats administered varying sulforaphane doses, the ganglion cell thickness approached normal levels. Concurrently, the production of pro-inflammatory cytokines TNF-α, IL-6, and IL-1β significantly diminished, while the activity of antioxidant enzymes GSH and SOD was augmented. Sulforaphane facilitated the nuclear accumulation of Nrf2, amplifying the mRNA expression of two crucial antioxidants downstream of Nrf2, namely heme oxygenase-1 (HO-1) and NAD(P)H quinone oxidoreductase 1 (NQO1), within the damaged retina. The Nrf2 activation under high-glucose conditions inhibited the inflammation, ROS production, protein kinase C, and other pathways, thereby safeguarding the cells from the oxidative stress in humans in vivo. Furthermore, sulforaphane demonstrated its ability to shield the retinal photoreceptor cells [209]. Studies indicate that sulforaphane may suppress the AGE-RAGE axis and inflammatory response by the MAPK pathway and NF-kB [212].

In the field of the clinical studies concerning the employment of antioxidants to treat DR, there has been a growing interest in testing combined antioxidant therapy [213]. For example, an investigation conducted by Garcia-Medina and associates in 105 diabetic patients with non-proliferative DR, with a followup period of 5 years, evaluating an antioxidant supplementation with lutein, vitamin C, alpha-tocopherol, niacin, beta-carotene, zinc, and selenium, showed a delay in the DR progression but no effect on the visual acuity [214].

In a 6-month randomized controlled clinical trial, Chous and colleagues administrated a combination of vitamins C, D3 and E (d-alpha tocopherol), zinc oxide, eicosapentaenoic acid, docosahexaenoic acid, alpha-lipoic acid (racemic mixture), coenzyme Q10, mixed tocotrienols/tocopherols, zeaxanthin, lutein, benfotiamine, N-acetyl cysteine, grape seed extract, resveratrol, turmeric root extract, green tea leaf, and pycnogenol to 70 patients with diabetes (NCT01646047), reporting an improvement in visual acuity. However, no changes in retinal thickness were detected [215].

Remarkably, Lafuente and co-workers tested a combination of anti-VEGFs with antioxidants in 55 patients afflicted by diabetes to manage diabetic macular edema for 3 years, describing a lower macular thickness in the supplement group when compared to the control group [216].

A recent systematic review by Alfonso-Muñoz et al. on the use of oral antioxidant supplementation to treat DR in humans has concluded that improved clinical outcomes are only observable after long followup periods, in terms of delaying the onset or reducing the progression of DR [217]. Hence, the effectiveness of an antioxidant supplementation on the visual acuity and macula edema in DR requires further investigation in the medium term, while antioxidants display an early impact on the retinal function parameters even after short followup periods, suggesting that they may be considered valuable prophylactic adjuvant agents in the early stages of DR [217].

Collectively, according to the current literature and considering the encouraging preclinical and clinical studies, antioxidants may be considered valuable supplements to treat DR and for avoiding complications and the progression of the disease. However, their efficacy appears to manifest as a particularly slowly progressive improvement in terms of the visual patient outcomes.

#### 3.2.4. Growth Factors and Other Therapies

Growth factor therapy emerges as a promising avenue in the treatment of DR. Beyond the well-known VEGF, nerve growth factor (NGF) plays a crucial role in fostering nerve cell growth and survival, potentially shielding against the neurodegenerative changes in DR, and therefore potentially preventing the occurrence and progression of the disease [218].

Insulin-like growth factor-1 (IGF-1) exhibits pro-angiogenic and photoreceptor protective effects in ocular lesions [219]. A recent study revealed that IGF-1 stimulates retinal cell proliferation via the activation of multiple signaling pathways, such as the PKC, MAPK, and phospholipase C cascades [220]. Reduced levels of IGF-1 have been observed in the eyes of DR patients, suggesting that exogenous IGF-1 supplementation could be a strategy to restore normal retinal function [221].

Moreover, established antidiabetic therapies, such as glucagon-like peptide 1 receptor agonists (GLP1RAs), have demonstrated benefits for DR. These therapies have demonstrated to reduce neurodegeneration, inflammation, and oxidative stress [10]. Additionally, GLP1R activation contributes to a decrease in the breakdown of the blood–retina barrier and angiogenesis [222]. The application of sodium glucose cotransporter 2 (SGLT2) inhibitors also presents an intriguing therapeutic avenue for DR. By reducing pericyte swelling through the inhibition of sodium-dependent glucose entry and diminishing the overexpression of the extracellular matrix, these inhibitors may preserve microvascular perfusion and prevent retinal ischemia [223]. In a diabetic mouse model, the SGLT2 inhibitor empagliflozin demonstrated the inhibition of oxidative stress, apoptosis, and the ability to restore tight junctions in diabetic retinas, highlighting the protective effects of SGLT2 inhibitors in DR [224].

### 3.3. Gene and Cell Therapy Advancements

In contemporary DR research, gene therapy strategies are directed towards modifying the pathological environment of the retina by regulating the expression of VEGF and TNF-α [225]. Alternatively, these strategies involve delivering therapeutic genes to retinal cells through viral vectors [226]. Promisingly, studies have showcased the potential of gene therapy in delaying or preventing retinal cell death, and potentially halting the progression of DR. For example, a gene therapy named AAV2-sFLT01 gene therapy, currently in clinical trials, seeks to block VEGF signaling and inhibit the formation of new blood vessels and retinal edema. By introducing a gene encoding this effect into the retina, this therapy aims to enhance the retinal neuron survival [227,228]. In the realm of the clinical trials testing gene therapy in vascular ocular disorders, important differences were observed in the INFINITY trial (NCT04418427), where the patients affected by diabetic macular edema and wet AMD treated with the AAV2.7m8 encoding aflibercept (ADVM-022) at the same dose ultimately displayed dose-limiting toxicity in DR, while encouraging results were reported in AMD, underscoring the detrimental impact of the diabetic condition on the ocular safety of AAV therapies [228,229].

Additionally, in the ALTITUDE clinical trial, while treating patients with DR without center-involved DME with a suprachoroidal injection of RGX-314, a molecule of AAV2/8 vectors encoding an anti-VEGF antibody fragment, the researchers obtained good tolerance at 6 months, with no drug-induced severe adverse events (NCT04567550; NCT05296447) [228,230]. The ALTITUDE trial is set to be completed in 2024, with the followup phase finishing in 2028 [228].

Collectively, gene therapy holds immense potential in DR treatment, but its safety, precision, and potential side effects require further verification.

Cell therapy represents another crucial avenue in DR treatment, involving the replacement of damaged retinal cells or stimulation of regenerative abilities through the transplantation of healthy retinal cells [231]. The treatment strategies aiming for neuroprotection and neuronal cell replacement have the potential to represent valuable alternative therapy options for halting the progression of DR [232]. Neuronal stem cell studies have emphasized the possibility of inducing the generation and replacement of the photoreceptors and retinal ganglion cells [233,234]. In fact, stem cells may replace dying neurons such as retinal progenitor cells, pluripotent stem cell-derived photoreceptors, and ganglion cells [231].

Stem cell therapy, particularly utilizing mesenchymal stem cells (MSCs) derived from bone marrow, has shown promising vascular effects in ischemic and diabetic murine models [235,236]. Mechanistically, MSCs release a wide range of growth factors, such as the NGF, so it is not surprising to report that the intravitreal injection of MSCs in the STZ-induced diabetic mouse model increased the ocular levels of neurotrophic agents, reduced the lipid peroxidation levels, and prevented ganglion cell loss [237].

In the field of the clinical research testing the human use of stem cells to treat DR, Gu and colleagues displayed an acceptable level of safety and efficacy regarding the intravenous injection of autologous bone MSCs in 34 eyes afflicted by DR. However, they did not report the optimal treatment window and infusion times for the therapy [238]. Currently, numerous clinical trials are underway to evaluate the efficacy and safety of stem cell therapy for human use [231,239,240]. These investigations focus on different types of stem cells, including endothelial progenitor cells (NCT01927315 and NCT03403283), induced pluripotent stem cells (NCT03403699), and autologous bone marrow stem cells (NCT01920867 and NCT01736059).

Nevertheless, employing stem cells to manage DR presents significant limitations and poses relevant challenges. First, the diabetic retina represents a hostile environment for homing cells like MSCs [239,240]. Second, specific types of stem cells, such as retinal progenitor cells, have a very limited capability to proliferate and differentiate. Therefore, it becomes crucial to identify the most suitable product for each individual patient to achieve successful treatment [231]. Third, translating the pre-clinical findings into clinical testing is challenging due to the imperfect characteristics of animal models in representing human environments [241].

## 4. Challenges and Limitations

The future research in the field of DR is poised to advance through various avenues, including a deeper exploration of the pathogenesis, the development of innovative diagnostic methods, the progression of novel treatment strategies, and the investigation of approaches for disease prevention. The recent investigations have revealed new biomarkers and examined fresh diagnostic and therapeutic modalities, enhancing our comprehension and management of DR. Recognizing that DR’s pathogenesis arises from multiple interacting factors, such as hyperglycemia, oxidative stress, inflammatory responses, and vascular endothelial cell damage, has been a pivotal stride. Notably, cytokines like VEGF and NF-κB play central roles in DR’s pathogenesis, and the identification of biomarkers like NGF, TNF-α, and glial cells holds promise for early DR diagnosis. Nevertheless, DR remains a significant visual impairment challenge globally for individuals with diabetes, underscoring the imperative to deepen our understanding and enhance the treatment strategies for this intricate disease.

Fundamental questions persist, such as why diabetes triggers DR in only certain patients and which genes and environmental factors influence the onset and progression of DR. These inquiries offer opportunities for a more complete comprehension of DR’s pathogenesis. The development of diagnostic methods remains an ongoing endeavor, with artificial intelligence-based fundus photo diagnosis systems showing promise but requiring enhancements in terms of the accessibility, accuracy, and usability of the underlying software and hardware facilities. Addressing key challenges, such as accurately predicting a patient’s DR risk and disease progression and creating precise and convenient large-scale screening tools, represents potential directions for future research.

While anti-VEGFs, antioxidants, anti-inflammatory drugs, and PKC inhibitory molecules have demonstrated effectiveness in mitigating DR in corresponding clinical trials, their utilization is significantly constrained by the high costs of the emerging therapies as well as the long-term safety profiles of the candidate drugs and the short- to mid-term efficacy of the antioxidant agents.

Despite being a primary treatment for DR, anti-VEGF therapy has limitations as it may not be suitable for all patients due to the necessity of long-term and recurrent intraocular injections, posing a burden. The safety and long-term efficacy of gene therapy require further investigation, while stem cell therapy faces challenges in obtaining an adequate quantity and quality of stem cells and ensuring the stable survival and functionality of the transplanted cells in the retina. Addressing the potential side effects poses a significant challenge for anti-inflammatory and other drug therapies. Moreover, the DR prevention strategies, heavily reliant on optimal blood sugar control, still encounter instances where patients develop DR despite meticulous blood sugar management.

## 5. Concluding Remarks

In summary, our review article has elucidated the molecular pathomechanisms underlying DR, presenting a comprehensive and up-to-date report on the emerging and experimental treatment strategies aimed at mitigating this debilitating and widespread disease. Future research into the pathogenesis must effectively integrate the data from various genes and environmental factors, while the novel diagnostic methods must prioritize precision and accessibility. The research and development of new therapeutic drugs and methods should emphasize safety, effectiveness, and clinical applicability. Furthermore, the DR prevention strategies must explore practical avenues for effective prevention. Each of these challenges demands substantial effort and resources in the future research, with a collective recognition that this ongoing process will be arduous. Continuous knowledge accumulation, theory updates, and technique enhancements are crucial for achieving a more profound understanding and improved treatment of DR.
